# Epidemiology, Risk Factors, and Prevention of Suicidal Thoughts and Behaviour in Prisons: A Literature Review

**DOI:** 10.5334/pb.1072

**Published:** 2021-11-22

**Authors:** Louis Favril

**Affiliations:** 1 Institute for International Research on Criminal Policy, Faculty of Law and Criminology, Ghent University, BE

**Keywords:** suicide, self-harm, ideation, action, vulnerability, stress

## Abstract

Suicide is a global public health concern that affects all echelons of society, albeit not equally so. Compared with adults in the general population, incarcerated offenders are at increased risk to consider, attempt, and die by suicide, which represents a substantial burden of morbidity and mortality in prisons worldwide. This review synthesises recent literature pertaining to the epidemiology, risk factors, and prevention of suicidal thoughts and behaviour among prisoners, and outlines a framework which emphasises the interplay between individuals (importation) and their surroundings (deprivation). The available evidence suggests that prison-specific stressors may exacerbate risk of suicide in an already vulnerable population characterised by complex health and social care needs. Emerging data point to differential mechanisms through which prisoners come to think about suicide and subsequently progress to suicidal behaviour. As risk of suicide is determined by a complex web of synergistically interacting factors, its management and prevention demands a cross-sectoral policy and service response that includes targeted interventions aimed at high-risk prisoners in combination with population strategies that promote the health and wellbeing of all people in prison.

Suicide is an important public health concern (Naghavi, 2019), currently ranking as the 15^th^ leading contributor to years of life lost (Vos et al., 2020). Throughout the world, over 700,000 individuals die by suicide every year (WHO, 2021), with millions more considering or attempting to do so (Borges et al., 2010). The lifetime prevalence of suicidal thoughts (suicidal ideation) and suicide attempt among adults in the general population is estimated to be 9% and 3%, respectively (Castillejos et al., 2021; Nock et al., 2008). Nearly one-third (29%) of people who think about suicide will at some point engage in suicidal behaviour, of whom 60% will do so within one year of ideation onset (Nock et al., 2008). Although research on suicidal thoughts and behaviour has proliferated in recent decades (Franklin et al., 2017), a synthesis of the literature is at times complicated by the multiple phenomena that are considered across the suicidal spectrum (Turecki et al., 2019; see ***Box 1*** for definitions).

**Box 1 Terms and definitions across the suicidal spectrum**

**Table:** 


Term	Definition
Suicide	Intentionally ending one’s own life.
Suicide attempt	Non-fatal self-injurious behaviour with inferred or actual intent to die.
Suicidal behaviour	Self-injurious behaviour that may result in ending one’s life, whether fatal (suicide) or not (suicide attempt). This term excludes suicidal ideation.
Suicidal ideation	Any thoughts about ending one’s own life, with or without a clear plan for suicide. Suicidal ideation is used interchangeably with suicidal thoughts.
Suicide risk	A composite term referring to one’s risk to consider, attempt, or die by suicide. This term includes both suicidal ideation and behaviour.
Self-harm	Non-fatal self-injurious behaviour with or without intent to die. This term does not distinguish between suicide attempt and non-suicidal self-injury.

Based on Turecki et al. (2019).

Suicidal thoughts and behaviour affect all echelons of society, albeit not equally so. Risk of suicide markedly varies across population groups, and disproportionately impacts the most vulnerable and disadvantaged members of society, including psychiatric patients and criminal offenders (Fazel & Runeson, 2020). In the population, 36% of suicide decedents have a lifetime history of justice contact (King et al., 2015) and 15% have ever been exposed to imprisonment (Fjeldsted et al., 2017). People involved in the criminal justice system—broadly defined—are 2 to 3 times more likely to die by suicide compared with their general population peers (Webb et al., 2011). Suicide *in prison* warrants particular concern, and its prevention has been highlighted as an international priority by the World Health Organization (WHO, 2007). Against this background, this structured review aims to provide a comprehensive synthesis of recent literature pertaining to the epidemiology, risk factors, and prevention of suicidal thoughts and behaviour among prisoners. Although juvenile (Borschmann et al., 2020) and community-based (Skinner & Farrington, 2020) offenders comprise high-risk groups for suicide as well, this review exclusively focuses on incarcerated adults.

## Epidemiology

Worldwide, some 11 million individuals (~95% of whom are men) are imprisoned at any given time, with 3-fold more people transitioning through prisons annually (Walmsley, 2018). Suicide is a leading cause of mortality in prisoners, accounting for around 30% of all prison deaths (Favril et al., 2019; Kaur et al., 2019; Rabe, 2012). Nationwide studies have consistently shown that prison suicide rates far exceed those in the general population, with rate ratios typically in the range of 3 to 9 (Duthé et al., 2014; Favril et al., 2019; Kaur et al., 2019; Morthorst et al., 2021; Opitz-Welke et al., 2013). In a cross-national study spanning 24 high-income countries, suicide rates in male prisoners were on average 4 times higher than those recorded in their age-equivalent community counterparts, with a higher proportionate excess in women (Fazel et al., 2017a). Nordic countries have the highest rates of prison suicide, followed by France and Belgium, where rates are more than 100 suicides per 100,000 prisoners (Fazel et al., 2017a). It should be noted, though, that official statistics may underestimate the true extent of the problem, owing to misclassification of deaths by suicide as undetermined causes or accidents (e.g., suicides by self-poisoning recorded as unintentional overdoses). About 90% of prison suicides are the result of hanging or self-strangulation (Favril et al., 2019; Gauthier et al., 2015; Humber et al., 2011b), which may reflect the limited availability of other methods for suicide inside prisons. In the United States, prisoners have a 33-fold higher odds of dying by hanging than do suicide decedents in the general population (Dixon et al., 2020).

Prisoners who take their own lives only represent the tip of the iceberg, as many more struggle with suicidal thoughts or engage in suicidal behaviour without a fatal outcome. Not only do suicidal ideation and suicide attempt constitute the strongest predictors of suicide in prison (Zhong et al., 2021), they equally represent important health outcomes in their own right that signal profound distress. A 1997 national survey of 3139 prisoners in England and Wales showed that 39% had experienced thoughts of suicide at some point in life and one-fifth (22%) had ever attempted suicide (Jenkins et al., 2005). More recent epidemiological studies in Australia (Larney et al., 2012), New Zealand (Favril et al., 2020a), and Belgium (Favril & O’Connor, 2021) have identified similar lifetime prevalence estimates for suicidal ideation (34–44%) and suicide attempt (19–22%) in randomly sampled prisoners, with higher rates in women than men (***Table 1***). In reference to community-based adults (Nock et al., 2008), suicidal thoughts and attempts thus appear to be 4 to 7 times more prevalent among incarcerated offenders, although direct comparisons are scarce (Jenkins et al., 2005). Furthermore, half (47–58%) of prisoners with suicidal thoughts go on to attempt suicide in their lifetime (Favril et al., 2020a; Favril & O’Connor, 2021; Larney et al., 2012), which is roughly twice the ratio (29%) documented among adults in the general population (Nock et al., 2008). When specifically considering *in-prison* outcomes, one in ten (9–13%) prisoners report having made a suicide attempt at some point during their incarceration (Dudeck et al., 2011; Encrenaz et al., 2014; Favril et al., 2020b; Ford et al., 2020; Sánchez et al., 2021). In the previous 12 months in custody, a quarter (24%) of prisoners have considered suicide (Favril et al., 2017). A total population study found the annual prevalence of self-harm (irrespective of suicidal intent) in English and Welsh prisons to be 5–6% in men and 20–24% in women, which greatly exceeds the less than 1% of adults in the general population who self-harm each year (Hawton et al., 2014).

**Table 1:** Lifetime prevalence (%) of suicidal ideation and suicide attempt in prisoners, by sex.


	SUICIDAL IDEATION			SUICIDE ATTEMPT		
	
	MEN	WOMEN	ALL	MEN	WOMEN	ALL

Australia	33	39	34	20	29	21

Belgium	43	58	44	20	37	22

England & Wales	38	54	39	22	40	22

New Zealand	34	43	35	19	28	19


Source: Favril et al. (2020a); Favril and O’Connor (2021); Jenkins et al. (2005); Larney et al. (2012).

It is clear that people in prison are at markedly increased risk to consider, attempt, and die by suicide relative to adults living in the surrounding community. What, now, might explain this disproportionate high risk of suicide?

## Risk factors

The excess risk of suicide in prisoners compared with their non-incarcerated counterparts has stimulated the scientific debate about possible explanations. Theoretical accounts have tended to fall within two main paradigms, which alternately focus on the individual prisoner and the prison environment (Dye, 2010; Favril et al., 2017; Liebling & Ludlow, 2016; Marzano, 2010). One line of clinical and public health research has highlighted the complex health needs and pre-existing vulnerability that prisoners ‘import’ into custody, typically set against a backdrop of social disadvantage, whose risk of suicide is equally heightened before and after incarceration. Another school of thought, mainly within sociology and criminology, has brought attention to the influence of the custodial environment by underlining the deprivations and ‘pains’ of imprisonment that precipitate suicide. These two—relatively isolated—bodies of literature attribute the causes of prison suicide either to background vulnerabilities that offenders bring with them (import) into prison or to factors that are specific to the prison environment (deprivations).

### Imported vulnerability

Before they are even incarcerated, prisoners comprise a vulnerable population at high risk of suicide. The life trajectories of offenders, on average, are characterised by socioeconomic disadvantage across a variety of indicators—most notably poor housing, low educational attainment, poverty, and abuse. As these entrenched inequalities that drive criminal justice involvement largely overlap with the social determinants of health (Caruso, 2017; Hughes et al., 2020; Marmot, 2018; Stewart et al., 2018), it is not surprising that people who pass through prisons are distinguished by poor health profiles (Dirkzwager et al., 2021; Fazel & Baillargeon, 2011; Kinner & Young, 2018). Many experience complex and co-occurring physical health conditions, such as infectious and chronic diseases (Dolan et al., 2016; Maruschak et al., 2015; Voller et al., 2016). In addition, meta-analyses have highlighted the excess prevalence of mental disorders in prisoners, including depression and psychosis (Fazel & Seewald, 2012), posttraumatic stress disorder (Baranyi et al., 2018), substance use disorders (Fazel et al., 2017b), and personality disorders (Fazel & Danesh, 2002). Taken together, it is clear that prisons accommodate a highly vulnerable group of individuals who face many complex health and social care needs.

Such markers of social disadvantage and health morbidity represent well-established risk factors for suicidal behaviour. A compelling body of evidence in the general population has demonstrated strong associations between risk of suicide and socioeconomic disenfranchisement, early-life adversity, trauma, physical illness, drug and alcohol abuse, psychiatric morbidity, and personality traits such as impulsivity and aggression (Carrasco-Barrios et al., 2020; Fazel & Runeson, 2020; O’Connor & Nock, 2014; Richardson et al., 2021; Turecki et al., 2019). These vulnerability factors not only contribute to risk of criminal justice involvement but, given their overrepresentation in prisoners, may also account for the disproportionate prevalence of suicidal thoughts and behaviour within custodial settings. Accordingly, the *importation model* argues that risk of suicide in prisoners is attributable to their social and health inequalities they bring with them (import) into prison. Evidence supporting this model comes from comprehensive meta-analyses (Favril et al., 2020c; Zhong et al., 2021) and systematic reviews (Lohner & Konrad, 2007; Marzano et al., 2016) which have consistently linked pre-prison vulnerabilities to increased risk of suicide in prison (***Table 2***). For example, prisoners with a prior history of self-harm are 7 times more likely than those without such a history to engage in suicidal behaviour while incarcerated (Favril et al., 2020c; Zhong et al., 2021). In addition, mental disorders are particularly strong risk factors for suicidal behaviour among prisoners, including mood, anxiety, psychotic, and substance use disorders (Favril et al., 2020c; Zhong et al., 2021), with comorbidity further compounding risk (Marzano et al., 2010; Rivlin et al., 2010). Borderline personality disorder is strongly associated with self-harm in prison, as opposed to antisocial personality disorder for which diagnostic criteria overlap with the reasons for entering prison (Favril et al., 2020c). Furthermore, excess risk of suicide in people committing violent crimes (e.g., murder and manslaughter) has been well established in the literature (Bukten & Stavseth, 2021; Webb et al., 2011; Zhong et al., 2021). It appears that the risks for both violent and suicidal behaviour coalesce in certain individuals, which could in part be neurobiologically determined (O’Donnell et al., 2015; Sahlin et al., 2017). Violent offenders may be characterised by high levels of impulsivity and aggression—traits known to increase risk of suicide (Marzano et al., 2011; Rivlin et al., 2013), with poor behavioural control being a likely mechanism. Childhood maltreatment is another robust risk marker for suicidal behaviour in prisoners (Angelakis et al., 2020; Favril et al., 2020c), pointing to a long-term susceptibility to suicide in people who have been exposed to trauma and abuse. Overall, these findings have been replicated in cross-sectional studies that focus on risk factors for suicidal ideation in large and unselected samples of prisoners (Favril et al., 2017; Jenkins et al., 2005; Larney et al., 2012; Sarchiapone et al., 2009). The elevated risk of suicide in offenders serving community sentences (King et al., 2015) and recently released prisoners (Haglund et al., 2014; Pratt et al., 2006) dovetails with the importation model, in that offenders’ vulnerability to suicide extends beyond the prison walls.

**Table 2:** Summary of models explaining risk of suicidal behaviour in prisoners.


MODEL	PREMISE	SELECTED RISK FACTORS*

Importation	Prisoners represent a non-random selection of vulnerable individuals who already are at high risk of suicide before imprisonment. The elevated risk of suicide in prisoners is a consequence of the social and health inequalities which they import into prison.	Socioeconomic disadvantage Trauma and childhood abuse Psychiatric (co)morbidity Drug and alcohol abuse Impulsivity and aggression History of self-harm

Deprivation	Prisoners are at increased risk of suicide by virtue of the highly demanding and restrictive environment they find themselves in. Deprivations and stressors inherent to the prison experience are what primarily account for the excess risk of suicide in prisoners.	Loss of freedom and autonomy Poor social support Lack of purposeful activity Solitary confinement Isolation and overcrowding Victimisation and bullying


* For a full overview of identified risk factors, see recent meta-analyses (Favril et al., 2020c; Zhong et al., 2021) and narrative reviews (Lohner & Konrad, 2007; Marzano et al., 2016).

In summary, the most vulnerable members of society, where levels of social disadvantage and health morbidity are most marked, are overrepresented within prisons. Justice-involved individuals already are at high risk of suicide before imprisonment due to histories of trauma, mental disorders, and substance abuse—intersecting vulnerabilities which they import into prison. From this perspective, risk factors operate the same in both prisons and the general population (***Table 2***). Under this essentially individual-centred take on the issue, the key limitation of the importation model is its narrow focus on background characteristics and pre-prison experiences of those who consider, attempt, or die by suicide, whilst principally ignoring the larger institutional context surrounding these outcomes. In doing so, the pains and harms produced by the prison milieu are largely overlooked, and therefore appears to suggest that imprisonment itself contributes little to risk of suicide in this vulnerable population.

### Prison stressors

There is little debate that incarceration is stressful in nature, and widespread concern has been raised over the detrimental health effects it might engender (e.g., Brinkley-Rubinstein, 2013; Goomany & Dickinson, 2015; Haney, 2012; Massoglia & Pridemore, 2015). In response to the constraints of the importation model, scholars have shifted focus towards the role of imprisonment itself in precipitating suicidal thoughts and behaviour. This perspective, known as the *deprivation model*, holds that prisoners are at high risk of suicide by virtue of the depriving and stressful environment in which they are detained (***Table 2***).

In support of this model, evidence has clearly shown that isolation, boredom, lack of purposeful activity, and victimisation (e.g., bullying and assault) while incarcerated all increase the likelihood of suicide in prisoners (Blaauw et al., 2001; Favril et al., 2017, 2020c; Huey & McNulty, 2005; Leese et al., 2006; Rivlin et al., 2013). Risk of suicide is found to be higher under social extremes of incarceration—being subjected to solitary confinement or, at the other end of the spectrum, living under conditions of overcrowding. As it inherently reduces protective factors such as meaningful social contact and access to purposeful activity, exposure to solitary confinement negatively affects prisoners’ mental health and confers an increase in risk of suicide (Brown, 2020; Haney, 2018; Luigi et al., 2020). Although this association could be confounded by individual characteristics of those who experience segregation (e.g., because of mental illness) and hence may reflect a selection effect, there is evidence that solitary confinement itself is an independent risk factor for suicidal behaviour (Frottier et al., 2007; Kaba et al., 2014), even *after* release from prison (Brinkley-Rubinstein et al., 2019). Furthermore, several ecological studies have reported on a positive relationship between overcrowding and suicide in prisons (Huey & McNulty, 2005; Leese et al., 2006; Rabe, 2012). A plausible explanation is that overcrowding places a substantial strain on the system and exacerbates problems in terms of prisoner-staff ratio, conflicts, and programming. Others, however, have observed a negative (Fritz et al., 2021) or null (Fazel et al., 2017a; van Ginneken et al., 2017) association, possibly owing to the protective effect of sharing a cell (Zhong et al., 2021). Indeed, the presence of a cellmate may mitigate risk by reducing feelings of social isolation through companionship and support, by limiting opportunities to engage in suicidal behaviour due to informal supervision, or both. Of note, national studies have shown that around half of all suicides in shared accommodation take place at a time when prisoners were alone in their cell—even though they were technically sharing one (Favril et al., 2019; Shaw et al., 2004). More generally, disconnection from family and friends on the outside appears to be a strong and consistent risk factor for suicidal thoughts and behaviour in prisoners (Favril et al., 2017; Jenkins et al., 2005; Rivlin et al., 2013), with subjective indices of social networks (perceived support) having a stronger association with suicide risk than do more objective measures (number of visits). Social support, or even just the belief that this is available, may be particularly salient to suicide risk in prisoners by virtue of the inherently isolated nature of incarceration.

Rates of suicide are not evenly distributed across types of facilities. For example, research suggests that suicide occurs more frequently in higher (compared to lower) security prisons (Bukten & Stavseth, 2021; Dye, 2010; Huey & McNulty, 2005; van Ginneken et al., 2017). This could reflect a difference in population composition with regard to individual risks (e.g., violent offenders) but it is likely that high-security facilities exemplify deprivation, isolation, and loss of control (van Ginneken et al., 2017). There also is evidence of spatiotemporal clustering of suicide (McKenzie & Keane, 2007) and self-harm (Hawton et al., 2014) within prisons. Furthermore, there are periods of imprisonment when risk is elevated—especially the early days and weeks following reception (Bukten & Stavseth, 2021; Favril et al., 2019; Humber et al., 2011b), during which deprivations are often experienced most intensely. Studies indicate that withdrawal from drugs or alcohol may amplify the risk of suicide shortly after arrival to prison (Dixon et al., 2020; Humber et al., 2011b; Radeloff et al., 2021). In a prospective cohort study, suicide risk was highest during the first week of custody, which after two months decreased among convicted but not remand prisoners (Hassan et al., 2011). Overall, prisoners on remand have a 4-fold increased risk of suicide relative to their convicted peers (Zhong et al., 2021), and suicides have been shown to differ between both groups as to timing and precipitating stressors (Konrad et al., 2007). The pre-trial period may be particularly challenging because of the sudden separation from family and friends, repeated court visits, and uncertainty regarding the future (Humber et al., 2013). First-time prisoners may feel especially anxious about facing an unknown situation and adjusting to a novel environment, which may drive them to contemplate suicide (Favril et al., 2017). Interfacility transfers, cell relocations, and changes in legal status (e.g., after sentencing or appeal) also comprise periods of heightened risk due to their stressful and disruptive nature (Favril et al., 2019).

In summary, there is compelling evidence that suicidal thoughts and behaviour are associated with institutional conditions and prison-specific stressors (***Table 2***). However, factors unique to the correctional milieu are unable to fully account for the excess risk seen in this population. The main critique on the deprivation model is that in similarly depriving conditions, or even within a single facility characterised by high levels of deprivation, the vast majority of prisoners will not consider, attempt, or die by suicide. As such, the deprivation perspective largely fails to explain why incarceration leads to suicide for some prisoners, but not others (Dye, 2010). This model assumes a monolithic prison population and by that overlooks prisoners’ individual needs and experiences that pre-date imprisonment.

### Combined model

Although its aetiology is not yet fully understood, the available evidence suggests that suicidal behaviour is rarely the consequence of a single factor or event, but rather depends on the cumulative and interactive effects of myriad biopsychosocial determinants (Fazel & Runeson, 2020; Franklin et al., 2017; Turecki et al., 2019). Accounts put forward to explain the excess risk of suicide in prisoners have traditionally been separated in individual (importation) versus environmental (deprivation) causes (***Table 2***). However, whilst empirical support was found for each model individually, both have important shortcomings, and scholars now agree that prison suicide is best explained by a combination of these two models (Dye, 2010; Favril et al., 2017; Marzano, 2010). This *combined model* moves beyond an either-or dichotomy and emphasises the dynamic interaction between individuals and their surroundings—in that prisons expose already vulnerable individuals to additional risk (Liebling & Ludlow, 2016). As noted by Favril and colleagues (2020c), prisoners import a predisposition to suicide into prison (characterised by social disadvantage, trauma, violence, and poor health) which interacts with prison-specific stressors (such as lack of purposeful activity, social and physical isolation, and victimisation). Key to this combined model is the notion that individuals respond differently to environmental stressors, largely as a function of their underlying vulnerability. In highly depriving environments or when faced with stressful events, prisoner vulnerabilities are more likely to be exposed. For example, vulnerable groups (e.g., sex offenders and people with mental illness or intellectual disability) disproportionately experience victimisation in prison (Fazel et al., 2016), thereby compounding risk in those already susceptible. Reciprocally, this model acknowledges that vulnerable individuals may successfully cope with imprisonment when conditions of confinement are less depriving (Dye, 2010).

Albeit relatively scarce to date, empirical attempts to bridge importation and deprivation models of suicide risk in prisoners largely support this theoretical notion. For example, ecological (Dye, 2010) and multilevel (Stoliker, 2018) analyses indicate that the combined effects of institutional conditions and prisoner characteristics best explain suicidal behaviour in prisons, more so than either component in isolation. In addition, findings from a case-control study support a model of suicidal behaviour in male prisoners which incorporates imported vulnerabilities and prison experiences, and underscores their interaction (Rivlin et al., 2013). Similarly, Marzano and colleagues (2011) suggest that trauma, poor social support, and mental health problems influence—and in turn are influenced by—women’s experiences of incarceration, and together contribute to suicidal behaviour in prison. Among women serving life sentences, pre-prison experiences and prison-related factors were both independently related to suicidal ideation while incarcerated (Dye & Aday, 2013), with similar findings observed in an unselected sample of male prisoners (Favril et al., 2017). Taken together, these data bolster support for a combined importation-deprivation model of prison suicide, which emphasises the exposure of vulnerable individuals to a stressful environment. This premise is consistent with a wider *stress-diathesis model* of suicidal behaviour, in which a predisposition (or diathesis) to suicide becomes heightened under the influence of environmental stressors (Mann et al., 1999; van Heeringen, 2001). The analogy is evident—the diathesis component pertains to imported vulnerability, whereas environmental stressors relate to prison deprivations. Such a dual framework (whether importation-deprivation or stress-diathesis) can account for why most prisoners (all exposed to a stressful environment) do not engage in suicidal behaviour, and why psychosocial vulnerabilities (which are overrepresented in prisoners) do not constitute a sufficient cause for suicide. In other words, “it cannot be argued that there is no psychiatric element in or predisposition to suicide in those who succeed, both in and out of prison; but what should be acknowledged is that just as outside, it is more usually a combination of (psychiatric) vulnerability, situational stress and individual perceptions which trigger the final suicide act than either component alone” (Liebling, 1992).

### From ideation to action

Although research has been influential in delineating a wide range of predisposing and precipitating risk factors for suicidal behaviour in prisoners (***Table 2***), it does not establish who is most likely to act upon their suicidal thoughts (Klonsky et al., 2021). Recent meta-analyses have identified suicidal ideation as the strongest of all risk factors for suicidal behaviour while incarcerated (Favril et al., 2020c; Zhong et al., 2021). This finding tallies with a process-oriented approach to suicide risk, in which suicidal ideation is considered an initial stage along the pathway towards suicidal behaviour (van Heeringen, 2001). Despite high relative risks, however, the absolute risk of suicide (Hubers et al., 2018) and suicide attempt (ten Have et al., 2009) following suicidal ideation is substantially lower. Evidence suggests that, while in custody, the large majority of prisoners will not translate their suicidal thoughts into action (Favril et al., 2020b; Lekka et al., 2006), which reflects a behavioural threshold (Favril & O’Connor, 2021). This implies that suicidal ideation is not a sufficient cause nor a sensitive risk marker for suicidal behaviour, and that people who *do* go on to attempt suicide may constitute a unique class of suicidal individuals. Along these lines, recent research in prisoners indicates that factors associated with suicidal ideation are different from those that govern the transition to suicide attempt, suggesting distinct underlying mechanisms (Favril et al., 2020a, 2020b). This is consistent with recent ideation-to-action theories of suicide, in which the development of suicidal thoughts and the transition to suicidal behaviour are conceptualised as distinct processes (Klonsky et al., 2018).

Among 1203 prisoners in Belgium, prison-specific stressors (pertaining to autonomy, safety, purposeful activity, and social support) and markers of poor mental health were found to contribute to the development of suicidal thoughts, but were not associated with the progression to suicide attempt in those with ideation (Favril et al., 2020b). Similarly, in a nationally representative sample of 1212 New Zealand prisoners, mental disorders were generally not associated with suicide attempt above and beyond their relationship with suicidal ideation (Favril et al., 2020a), which strongly concurs with epidemiological data in the general population (Borges et al., 2010; Nock et al., 2009, 2010). These findings collectively suggest that factors relating to the prison regime and psychiatric morbidity may affect the cognitive (ideation) rather than the behavioural (attempt) spectrum of the suicidal process. Instead, behaviours characterised by disinhibition (substance use, violent offending, and non-suicidal self-injury) were found to double the odds of suicide attempt among prisoners with suicidal ideation, as did exposure to trauma and suicidal behaviour of others (Favril et al., 2020a, 2020b). Deficits in executive functioning may reflect a shared diathesis underpinning these associations, in that individuals with reduced inhibition may have difficulty resisting the urge to act upon a suicidal desire (Saffer & Klonsky, 2018). In prisoners, drug use and aggression—directed towards others (violent offending) or oneself (non-suicidal self-injury)—are related to low impulse control (Bernstein et al., 2015; Carli et al., 2010; Meijers et al., 2017), which might explain their tendency towards behavioural enaction in the face of suicidal thoughts (Favril & O’Connor, 2021). Together, these data lend support to the hypothesis that behavioural disinhibition might act as a catalyst in the transition from thoughts to acts of suicide (e.g., Mann et al., 1999; Mars et al., 2019; Turecki et al., 2019).

In summary, emerging evidence highlights differential associations between risk factor domains and distinct stages along the suicidal process in prisoners (Favril et al., 2020a, 2020b). Whilst mental disorders and prison-specific stressors may drive prisoners to consider suicide, it appears to be the presence of an imported vulnerability characterised by behavioural disinhibition that primarily affects their propensity to translate suicidal thoughts into action.

## Prevention

The high risk of suicide in prisoners, and the substantial burden it exerts, underscores the need for evidence-based prevention (NICE, 2018; WHO, 2007). The literature outlined above entails two important implications for suicide prevention in prisons, which may also inform efforts to prevent suicide in other institutional settings (e.g., psychiatric inpatient units and immigration detention centres). First, recognising the interaction between the vulnerable individual and a challenging environment, there is a need for a comprehensive approach to suicide prevention that incorporates targeted strategies aimed at high-risk prisoners in combination with population strategies that address systemic and environmental stressors within prisons (Marzano et al., 2016). Second, recognising that the development of suicidal thoughts and the transition to suicidal behaviour reflect distinct processes with distinct predictors, it is vital to distinguish which interventions target the onset of suicidal ideation in the whole population and which are meant to impede progression to suicidal behaviour in people who are already thinking of suicide (Klonsky et al., 2021). Overall, in relation to the aims and targets of specific interventions, the focus on distinct outcomes (thoughts or behaviour) and their causes (individual or environmental) should be carefully taken into account when developing a prison policy to prevent suicide.

Effective suicide prevention rests upon early identification of high-risk individuals who would benefit from targeted interventions. To do so, suicide risk assessment should not be limited to a one-off event at the point of reception into prison, but must be an ongoing and systematic process at regular intervals throughout prisoners’ period of incarceration (Marzano et al., 2016). Although screening tools are recommended by the WHO (2007) to guide risk stratification and inform treatment allocation, few such scales have yet been developed specifically for use in prison populations (Gould et al., 2018) and further research is needed to test their reliability and predictive validity (Horton et al., 2018; Ryland et al., 2020). Once risk has been identified, a multidisciplinary management process should be established with clearly articulated procedures and policies outlining responsibilities for placement, monitoring, and treatment (Humber et al., 2011a). Successful multi-agency collaboration will be contingent upon good communication and information flow between services, and issues relating to professional confidentiality—what information can be shared under which circumstances—should be clarified in protocols (Senior et al., 2007; Slade & Forrester, 2015).

Prisoners at imminent risk of suicide should be placed under observation. Monitoring intervals (e.g., every 15 or 30 minutes) should match the level of risk and access to suicide means (e.g., ligature points) should be limited (Konrad et al., 2007). As a general principle, prisoners should be housed in the least restrictive accommodation which at the same time maximises their safety. Solitary confinement as a preventative measure should be avoided given its harmful potential. Whilst interventions aimed at the *physical* prevention of suicide may have the ability to save lives, they do not address the reasons why prisoners are suicidal in the first place, nor do they reduce the longer-term risk of suicide. Appropriate psychosocial care and support should be provided.

Mental health services need to be adequately resourced and linked with evidence-based interventions (Bolton et al., 2015; Fazel et al., 2016) to address the high level of unmet clinical need in prisoners (Jakobowitz et al., 2017; Tyler et al., 2019). International minimum standards for the treatment of prisoners, including the 2015 Mandela Rules adopted by the United Nations, should be adhered to (Forrester et al., 2018). Cognitive behavioural and mindfulness-based therapies currently have the most consistent evidence in reducing depression and anxiety among prisoners, albeit with modest effects (Yoon et al., 2017). A recent randomised controlled trial supports the (cost-)effectiveness of group interpersonal psychotherapy for prisoners with depression (Johnson et al., 2019). Trauma-informed care should also be considered, especially interventions which are tailored to the unique needs of incarcerated women (Bartlett et al., 2015; Malik et al., 2021).

In addition to mental health care to treat underlying psychiatric conditions, psychosocial interventions that *directly* target suicidal thoughts and behaviour should be made available to prisoners in need (Meerwijk et al., 2016). Current evidence, however, largely relies on either small and uncontrolled prison studies (Winicov, 2019) or trials conducted within community settings (Fox et al., 2020; Witt et al., 2021), although recent randomised controlled trials found encouraging results that cognitive behavioural therapy (Pratt et al., 2015) and psychodynamic interpersonal therapy (Walker et al., 2017) may improve outcomes in suicidal prisoners. Because this work is still only at the piloting stages, further adequately powered trials are required to evaluate their effectiveness in prisons. Furthermore, mounting evidence indicates that self-guided digital interventions (Torok et al., 2020) and brief interventions such as safety planning (Doupnik et al., 2020; Nuij et al., 2021) are promising to prevent suicidal ideation and attempt, respectively, in the general population, which present scalable options for treatment in prisons. However, because interventions developed in the community cannot be simply translated to prisons, these should be tailored to the complex needs of prisoners and adapted to suit the demands of the prison context.

Although strategies that focus on detecting, managing, and treating suicidal prisoners comprise a central component of any prevention policy, these alone will not be sufficient. Interventions should equally address the onset of suicidal thoughts by favourably shifting risk and protective factors across the entire prison population, rather than in specific groups of prisoners who are considered to be at risk. Within a ‘healthy prison’ approach to suicide prevention, targeted interventions should be supplemented by population strategies that promote the health and wellbeing of all people who are incarcerated, and mitigate any detrimental effects of imprisonment. Such environmental interventions and changes to the prison regime should address aspects of safety, autonomy, purposeful activity, and social support (Favril et al., 2017; Marzano et al., 2016). For example, providing prisoners with sufficient opportunities to take part in constructive out-of-cell activities (e.g., recreation, education, work, and physical exercise) can reduce suicide risk and benefit mental health more generally (Stephenson et al., 2021). This approach is further supported by neuropsychological evidence indicating that active prison regimes contribute to positive outcomes in prisoners (Meijers et al., 2018). Prisoners should also be able to maintain meaningful contact with their family and friends on the outside through social visits, and video conferencing could be a feasible option to overcome logistical barriers for social interaction. In addition, enabling access to trained ‘buddies’ or ‘listeners’ through peer-based support schemes (Bagnall et al., 2015) and promoting frontline staff to have regular contacts with prisoners (Ludlow et al., 2015) might be beneficial to integrate social support in suicide prevention efforts. Qualitative research inquiring into the experiences of prisoners who have attempted suicide suggests that being able to talk to staff—and being listened to—is key to suicide prevention (Borrill et al., 2005; Marzano et al., 2016; Suto & Arnaut, 2010).

In prisons, certain aspects of the closed and restricted environment can make suicide more easily preventable than in the community (through closer monitoring and means restriction), whereas institutional stressors and deprivations (such as social isolation and lack of purposeful activity) may exacerbate risk of suicide in an already vulnerable population by virtue of their intersecting health and social care needs (Marzano et al., 2016). As no singular approach will likely be able to significantly impact on an issue as complex as suicide, prevention efforts should be integrated and synergistic (Hofstra et al., 2020). Thus, in order to address both predisposing and precipitating risk factors, suicide prevention will require a multilevel approach that combines population and targeted strategies (Barker et al., 2014). National standards and guidelines covering best practice (Daigle et al., 2007) should be developed or updated based on the latest evidence, though one size will not fit all—the considerable variability between prisons (and wings) necessitates that policies are tailored to the specific setting and local needs. Overall, effective management and prevention will be contingent on commensurate coordinated policy and practice efforts that emphasise cross-sectoral collaboration between health, social care, and criminal justice agencies. Continued research should inform policy and practice to achieve the public health objective of reducing preventable mortality in people who at some point experience incarceration.

## Conclusion

Suicide is a global public health concern that disproportionately affects the most vulnerable and disadvantaged members of society. Suicidal thoughts and behaviour are common in prisoners, contributing to substantial morbidity and mortality, with a wide range of modifiable risk factors. Key to understanding and preventing risk of suicide in prisoners is the interplay between individuals and their surroundings. Emerging evidence highlights differential associations between risk factor domains and theoretically distinct points along the suicidal process; prison-specific stressors increase the likelihood of developing suicidal thoughts, whereas imported vulnerabilities characterised by behavioural disinhibition facilitate the transition towards suicidal behaviour. Given that risk of suicide is determined by a complex web of synergistically interacting factors, there is a need for a comprehensive approach to suicide prevention that incorporates both targeted interventions and population strategies, with multi-agency collaboration having a key role.
